# Tyrosine Kinase Inhibitors Available for Chronic Myeloid Leukemia: Efficacy and Safety

**DOI:** 10.3389/fonc.2019.00603

**Published:** 2019-07-03

**Authors:** Valentín García-Gutiérrez, Juan Carlos Hernández-Boluda

**Affiliations:** ^1^Servicio de Hematología, Hospital Universitario Ramón y Cajal, Instituto Ramón y Cajal de Investigación Sanitaria (IRYCIS), Madrid, Spain; ^2^Servicio de Hematología, Hospital Clínico Universitario, Institute of Health Research (INCLIVA), Valencia, Spain

**Keywords:** chronic myeloid leukemia, treatment, safety, efficacy, patients

## Abstract

Chronic myeloid leukemia (CML) is currently a disease in which patients can enjoy a near normal life-expectancy. However, since the majority of patients will need to remain on treatment indefinitely, physicians in care of CML patients need be familiar with the indications and toxicities of all approved tyrosine kinase inhibitors (TKI). In clinical practice, there are five TKI (imatinib, nilotinib, dasatinib, bosutinib, and ponatinib) that are available in different scenarios and have distinct safety profiles. Decisions regarding first line treatment must be based on CML risk, comorbidities, and patients expectations. Despite the excellent outcome, half of the patients will eventually fail (due to intolerance or resistance) to first line treatment, with many of them requiring a third or even further lines of therapy. When selecting for such patients, it is essential to distinguish between failure and intolerance to previous TKIs. In the present review, we will address all these issues from a practical point of view.

## Introduction

The prognosis of chronic myeloid leukemia (CML) has changed during the past two decades from a disease with an overall survival (OS) of 5 years only to one in which patients can enjoy a near normal life-expectancy (1, 2). Such remarkable improvement in the patients' outcome is mainly due to the introduction of imatinib into the clinic (the first approved tyrosine kinase inhibitor [TKI]), but also to the approvals of others TKIs. Currently, there are five TKIs available for CML treatment in clinical practice (Table 1) (3). Although a proportion of patients (around 20%) (4) will be able to successfully discontinue TKI treatment after achieving a deep molecular remission, most of them will require to keep on treatment indefinitel (5). In this scenario, it is crucial for physicians caring for CML patients to be aware of which TKIs are available for each particular clinical situation, what can be expected from them, and how to manage their potential side effects. In the present review, we will briefly address these issues from a practical point of view.

**Table 1:** Tyrosine kinase inhibitors approved for patients with chronic myeloid leukemia (eMC 2019).

	**Chronic phase**		**Accelerated phase**	**Blastic phase**
	**First line**	**Second or later treatment lines**		
Imatinib	Approved	After failure of interferon	Approved	Approved
Dasatinib	Approved	Resistance to prior TKIs including imatinib	Approved	Approved
Nilotinib	Approved	Resistance or intolerance to prior therapy including imatinib	Approved	Not approved
Bosutinib	Approved	Previously treated with one or more TKIs and for whom imatinib, nilotinib, and dasatinib are not considered appropriate treatment options	Approved	Approved
Ponatinib	Not approved	Resistance or intolerance to dasatinib or nilotinib and for whom subsequent treatment with imatinib is not clinically appropriate; or with the T315I mutation	Approved	Approved
Radotinib^a^	Approved	Approved	Not approve	Not approve

a *Radotinib is only approved in Korea*.

## Efficacy of TKIs in Different Clinical Scenarios

### TKI Treatment in Newly Diagnosed Chronic Phase CML Patients

#### Imatinib

Imatinib was the first TKI approved for the treatment of CML on the basis of a high level of activity in phase 2 studies including patients who were resistant or intolerant to interferon (6). With regard to the frontline therapy, in the IRIS study, the estimated rate of complete cytogenetic response (CCyR) at 12 months in the imatinib arm was 69%. Such responses translated into a superior progression free survival (PFS) (primary end point of the study) in patients treated with imatinib as compared to those assigned to interferon and cytarabine (the standard of care at that time), with this leading to an early approval of imatinib in the first line setting in 2002 (7). Last update of the IRIS study showed an estimated OS rate of 83% at 10 years (20.1% of patients had unknown survival status when data was analyzed). It should be mentioned that, despite these excellent results, 31 and 52% of patients assigned to imatinib discontinued treatment by 5 and 10 years of follow up, respectively. The main cause of treatment discontinuation was the unsatisfactory therapeutic effect (11%), while only 4% of patients discontinued treatment due to side effects (8). Results from real life experiences studies have shown superior responses compare to data from clinical trials (9).

The 400 mg daily dose for imatinib was based on data from the early phase 2 study demonstrating adequate efficacy and tolerability (10). However, the optimal dose of imatinib in chronic phase (CP) CML regarding the efficacy has not been established. To this respect, several studies have evaluated higher (600 or 800 mg daily) imatinib doses in different clinical scenarios. Liu et al conducted a meta-analysis showing the benefit of imatinib higher doses in terms of CCyR, but with increased toxicity. Of note, higher imatinib doses were not associated with any improvement in the rate of disease progression to advanced phases nor in the OS (11).

Dasatinib, nilotinib, and bosutinib are second generation TKIs (2GTKI) initially approved in CML patients who were resistant or intolerant to imatinib. Due to a more potent *in vitro* inhibition of the unmutated BCR-ABL kinase with a good safety profile, these 2GTKIs were later evaluated and approved in the first line setting (10).

#### Dasatinib

Dasatinib as frontline treatment in CP CML was first evaluated in an exploratory single-institution trial showing CCgR rates of 98% by 12 months (12). Later on, a randomized prospective study (the Dasision trial) compared dasatinib 100 mg QD vs. imatinib 400 mg QD in newly diagnosed CP CML patients. After 12 months of treatment, dasatinib showed higher rates of confirmed CCyR (primary endpoint of the study) and MMR, which led to the approval of dasatinib for the upfront treatment of CML in CP (13). Final update of this study, with 5 years follow up, showed that 61 and 63% of the patients remained on dasatinib and imatinib, respectively. Main reasons for treatment discontinuation were lack of efficacy (14 vs. 11% for imatinib and dasatinib, respectively) and intolerance (16 vs. 7% for dasatinib and imatinib, respectively). The use of dasatinib was associated with higher rates of deep molecular responses (MR4 and MR4.5) (14), which could eventually translate in more candidates to TKI discontinuation studies. However, no differences in PFS or OS between the imatinib and the dasatinib arms were observed. A recent study has evaluated the use of dasatinib 50 mg daily in 75 newly diagnosed CP CML patients. At 12 months, 79, 71, and 46% of the patients achieved a MMR, MR4, and MR4.5 respectively, with only one patient developing pleural effusion (15).

#### Nilotinib

Nilotinib 400 mg bid was initially tested in a phase 2 trial conducted by the GIMEMA group including 73 newly diagnosed CP CML patients. Remarkably, CCyR at 12 months (primary endpoint of the study) and MMR were 96 and 85%, respectively (16). After 6 years follow up, 75% of patients remained on nilotinib with a cumulative incidence of MR4 of 76% (8). Another phase II clinical trial performed at the MDACC evaluated the use of frontline nilotinib 400 mg bid showing probabilities of CCyR and MMR at any time of 98 and 76%, respectively (17). Frontline nilotinib was later approved based on the ENESTnd trial (a company sponsored phase 3 randomized prospective trial) that compared two nilotinib regimes (300 and 400 mg bid) with imatinib 400 mg in newly diagnosed CML patients. Both nilotinib arms (300 and 400 mg bid) showed higher probabilities of MMR (primary end point of the study) and CCyR. A better toxicity profile was observed in the nilotinib 300 mg bid arm and this dosage was therefore chosen for registration in the first line setting (18). By 5 years, more patients achieved MMR in the nilotinib arm (77 vs. 60% for nilotinib 300 mg bid and imatinib 400 mg, respectively) and MR4.5 (54 vs. 31% for nilotinib 300 mg bid and imatinib 400 mg, respectively). After 5 years follow up, 59 and 49% remained in the nilotinib 300 mg bid and imatinib arms, respectively. Both nilotinib regimens had lower rates of transformation to advanced phases than the imatinib arm, but again, no difference in OS was found (19).

#### Bosutinib

Bosutinib is a dual Src/Abl TKI with minimal inhibitory activity against c-KIT or platelet-derived growth factor receptor, non-specific targets potentially associated with toxicities reported with other 2GTKIs (20). Bosutinib demonstrated significant clinical activity in patients with CP CML who had resistance or intolerance to prior TKI therapy (21, 22). Bosutinib was subsequently tested in newly diagnosed CP CML patients in the BELA trial (imatinib 400 mg QD vs. bosutinib 500 mg QD). Although bosutinib treatment was associated with a higher probability of achieving MMR than imatinib, no differences in CCyR (primary endpoint) were observed, and bosutinib 500 mg was therefore, not approved for this indication. In this trial, an unexpectedly high number of patients (48% of total) discontinued bosutinib treatment due to an AE (23). Later on, bosutinib 400 mg QD was compared to imatinib 400 mg QD in another randomized prospective phase III clinical trial (the BFORE study). Bosutinib 400 mg was associated with a higher probability of achieving MMR (primary endpoint of the study), CCyR, and deep molecular response by 12 month (24). Based on these results, bosutinib 400 mg was finally approved in CP CML newly diagnosed patients.

#### General Considerations for the Frontline Treatment of CP CML

Currently, imatinib, nilotinib, dasatinib, and bosutinib are approved in newly diagnosed CP CML patients. All 2GTKIs have demonstrated higher probabilities of CCyR and MMR than imatinib, while which are well-known surrogate endpoints for PFS and OS. However, none of the 2GTKIs has shown to increase OS when compared to imatinib. The reason for this finding is most likely due to the effectiveness of second line TKI treatment in imatinib resistant patients. On the other hand, each TKI has a different toxicity profile which should be taken into account at the time of treatment selection, since the incidence and tolerability of some side-effects may depend on the patient's comorbidities (25). Another relevant point to consider is the recent introduction of generic imatinib in clinical practice, which has markedly decreased the price of treatment (26, 27). Different groups have identified how molecular response at 3 months can identify patients in risk of progression, and therefore, an early treatment intervention could improve outcomes in imatinib treated patients (28). In view of the abovementioned factors, imatinib, at this moment, is the drug most commonly used as frontline treatment in clinical practice. Having said that, there are some situations in which the frontline use of a 2GTKI deserves consideration. First, 2GTKIs (dasatinib and nilotinib) have demonstrated to decrease the risk of disease progression in patients with intermediate or high risk groups by the conventional scoring systems [Sokal in the ENESTnd (19) and Hasford in the DASISION study (14)]. Hence, 2GTKI are the preferred option for the first-line treatment in patients with intermediate or high risk scores according to the NCCN (29) and ESMO guidelines (30). Second, treatment discontinuation is a new treatment goal in CML, particularly in younger patients (31, 32). The use of 2GTKIs has been associated with higher rates of DMR, which are usually achieved significantly faster than with imatinib (14, 19, 24). Consequently, 2GTKIs could potentially increase the number of patients that are able to discontinue treatment and, importantly, diminish the duration of treatment before discontinuation (33). In this line, a recent retrospective study from the Italian group has shown higher treatment free remission (TFR) rates in patients treated with 2GTKIs as compared to imatinib (34). The potential benefit of using a 2GTKIs in first line CML patients with the goal of TFR is currently being evaluated in a prospective clinical trial (Sustrenim Study NCT02602314). Lastly, it should be reminded that nilotinib, based on two discontinuation studies, is the only TKI that has been approved for the TFR label. However, once a patient achieved and maintained a deep molecular response, with whatever TKI, treatment discontinuation may be considered (29, 30).

### TKI Treatment After Failure to Imatinib in CP CML Patients

Approximately half of patients who receive frontline imatinib will eventually fail after 5 years of treatment (14, 19). Reasons for imatinib failure are similarly distributed into resistance and intolerance to treatment. Dasatinib and nilotinib are approved in patients failing to imatinib based on two company sponsored phase 2 trials. Bosutinib is also indicated in Europe after imatinib failure, but only in those patients in whom dasatinib and nilotinib are not considered adequate treatment options (5). Overall, all three 2GTKIs showed similar efficacy, with probabilities of obtaining CCyR around 50% (Table 2). All TKIs demonstrated higher rates of response in intolerant patients than in resistant ones (35–37). A recent study real life setting has shown how up to 70% of patients achieved CCyR with similar responses rates between dasatinib and nilotinib (38).

**Table 2:** Treatment responses with 2GTKI in patients with imatinib failure.

	**Dasatinib 100 mg**		**Nilotinib 400 mg bid**		**Bosutinib 500 mg qd**	
	**Resistance**	**Intolerance**	**Resistance**	**Intolerance**	**Resistance**	**Intolerance**
**Follow up**	**24 months**		**24 months**		**24 months**	
CCyR^a^	44%	67%	41%	51%	48%	52%
MMR^b^	37%		28%			
PFS^c^	80%		64%		79%	
OS^d^	91%		87%		92%	

a *Complete cytogenetic response*.

b *Major molecular response*.

c *Progression free survival*.

d *Overall survival*.

New treatment strategies have also been evaluated in patients not classified as “classical failures” by European LeukemiaNet (ELN) recommendations. In this sense, the lack of early molecular response (defined as MR > 10% at 3 months) has been associated with a worse prognosis in terms of PFS and OS (5, 29, 30). Whether this early molecular response should be considered as hallmark defining failure patients has been debated during last years. One reason for not considering patients without early molecular response as failures was the absence of data demonstrating that a treatment change could improve the results. In this sense, a recent phase III clinical trial has demonstrated a beneficial effect of changing treatment to dasatinib in this group of patients (39). The results of a treatment change to 2GTKIs (dasatinib and nilotinib) have also been evaluated in patients with responses classified as “warning” by the ELN recommendations (previously called “suboptimal responders”). The Lasor study compared the strategy of a treatment change to nilotinib 400 mg bid vs. imatinib 600 mg in CML patients with suboptimal response. Patients who changed to nilotinib had better rates of MMR and deeper responses, but 20% of them had to discontinue nilotinib due to side effects without a benefit in PFS (40). The Dasapost study evaluated the results of a treatment change to dasatinib in patients with late suboptimal response (CCyR without MMR), showing 66% probabilities of obtaining MMR (41). The ENESTcmr study evaluated the results of changing to nilotinib 400 mg bid in imatinib treated patients who had not achieved MR4. Treatment change was associated with a higher probability of DMR. However, a significant proportion of patients (12%) on the nilotinib arm experienced a cardiovascular event, probably related to the nilotinib dose and the inclusion of an unselected population with baseline cardiovascular risk factors (42). Finally, the Enestpath trial is currently evaluating the feasibility of TFR with nilotinib 300 bid in patients not achieving DMR with imatinib. Preliminary results have showed similar probabilities of DMR with a lower incidence of cardiovascular events compared to the ENESTcmr study (43).

It is of note that the approved doses for second-line treatment with nilotinib and bosutinib, 400 and 500 mg QD, respectively, are higher than those used for first-line treatment. Since higher doses of these drugs are commonly associated with side effects, the use of lower doses could be an interesting option for imatinib intolerant patients. To this respect, nilotinib 300 mg bid has been evaluated in imatinib intolerant or imatinib treated patients with persistent low-grade side effects. Treatment changed to nilotinib translated in benefits in terms of responses, safety profile with an improvement in quality of live (QoL) (44, 45). The use of lower bosutinib doses is being explored in clinical trials (NCT02906696 and NCT02810990). Dasatinib was first approved at the dose of 70 mg bid based on data from the START-R trial. However, additional evidence from dose-optimization studies resulted in the current recommended dose of dasatinib (100 mg/day) for CP CML patients failing to imatinib (35).

#### General Considerations for the Second-Line Treatment After Imatinib Failure

Overall, nilotinib, dasatinib, and bosutinib have all demonstrated similar response rates in this clinical setting. Since mutations in the BCR-ABL kinase domain constitute the main known cause of resistance to imatinib treatment and some mutations have different sensitivities to 2GTKI, it is mandatory to study the mutational status to select the best treatment option in imatinib resistant patients (5). All 2GTKIs are approved treatment options for the minority of patients that progress to the accelerated phase of CML while on imatinib, while dasatinib, and bosutinib are the only ones approved for patients developing a blast crisis after imatinib treatment. Treatment change to 2GTKIs in patients classified as “warnings” has been associated with an improvement in MMR and DMR rates. However, since this strategy could lead to the appearance of new adverse events, the indication of treatment change should be individualized. Those patients without a DMR motivated for TFR may be considered for treatment changed with the goal of discontinuation therapy (4).

Once again, the toxicity profile of each TKI and the patient comorbidities should be considered at the time of selecting treatment. This is particularly relevant to prevent the development of cross intolerance (which means that patients can suffer the same type of toxicity that led to TKI treatment discontinuation in the first place), although this complication is uncommon. A *post-hoc* analysis of the pivotal phase 2 study with nilotinib as second line therapy showed that only one among 75 (1%) patients with non-hematologic imatinib intolerance experienced a similar grade 3/4 adverse event (AE), whereas 3 (4%) experienced a similar persistent grade 2 non-hematologic AE (46). The overall incidence of cross intolerance to bosutinib was 7.0% in patients who discontinued imatinib due to a non-hematologic AE, with incidences of 4.8, 7.7, and 0% for patients who discontinued due to rash, diarrhea, or edema, respectively (47).

### Treatment Options After Failure to 2GTKI Therapy

Although a significant proportion of patients respond to 2GTKIs therapy after imatinib failure, most of them (70%, approximately) will eventually discontinue such treatment in the short-term due to loss of response or toxicity (35–37).

The use of third line nilotinib after dasatinib failure was evaluated in a small phase II clinical trial that included 37 CP CML patients. After 12 months of treatment, 24% of them achieved CCyR, with only 54% remaining on the study (48). In a phase 1/2 study of bosutinib in third-line therapy or later, the probability of newly attained CCyR was 26%, and after 4 years of follow-up, only 24% of patients were still on treatment (49). The Spanish CML Group (GELMC) evaluated the use of bosutinib in patients with prior failure to dasatinib and nilotinib. While the probabilities of obtaining CCyR and MMR in resistant patients were only of 25 and 14%, respectively, they increased up to 94 and 42%, respectively, in intolerant patients (50).

Ponatinib is a third-generation TKI approved in CML patients with refractory CML or Philadelphia chromosome–positive acute lymphoblastic leukemia (Ph + ALL) and those harboring the BCR-ABL1T315I mutant. The PACE trial evaluated ponatinib 45 mg QD in 267 CP CML patients of whom >90% had previously received two or more TKIs (i.e., at least two of the following: imatinib, dasatinib, nilotinib, and bosutinib). Despite such population of heavily pretreated patients, 56% of them met the primary end point of achieving a major cytogenetic response (MCyR) by 12 months. By 5 years follow up, 70% of patients harboring the T315 mutation achieved a CCyR. Moreover, the responses occurred rapidly, at a median time of 2.8 months from treatment initiation. Twelve-month estimates of PFS and OS in CP-CML patients were 80 and 94%, respectively (51). After 5 years of follow up, 54 and 40% of the patients achieved CCyR and MMR, respectively. Responses were durable, with 82 and 59% of those who achieved MCyR by 12 months and MMR at any time, respectively, maintaining responses at 5 years. Current recommendations of dose reductions (in order to avoid vascular events) in ponatinib responded patients seem not to compromise previous responses (52).

Only a minority of CP CML patients (10–15%) have resistance to frontline treatment with a 2GTKIs during the first year of follow up (53). In this setting, retrospective studies have shown that 22 to 26% of patients can achieve a CCyR by changing the type of 2GTKIs (dasatinib for nilotinib and viceversa (54). The use of second line ponatinib in patients who received 2GTKIs as first line therapy (nilotinib or dasatinib) due to primary or secondary resistant has been evaluated in a retrospective study. Of note, 85% of patients improve baseline responses with no appearance of thrombotic events (55). Interesting, up front low ponatinib dose (15 mg) has been shown as an effective and safety strategy in patients resistance/intolerant to previous TKIs (56, 57).

#### General Considerations for the Management of Patients After 2GTKI Failure

In patients unable to tolerate a 2GTKI, the use of an alternative 2GTKI based on the patient's comorbidities and previous side effects seems reasonable. In patients with resistance to a prior 2GTKI, ponatinib seems to be the TKI that offer the highest rates of response. In this sense, a matching-adjusted indirect comparison of third-line ponatinib (*n* = 70) vs. bosutinib (*n* = 119) showed CCyR rates of 61 vs. 26% with an estimated probability of maintaining CCyR at 4 years of 89 vs. 54%, respectively. Discontinuation rates due to death, disease progression or unsatisfactory response were 9% with ponatinib and 42% with bosutinib (58). An alternative treatment option for 2GTKI resistant patients is allogeneic hematopoietic stem cell transplantation. A recent publication has compared the use of ponatinib and transplantation in patients harboring the T315I mutation who failed 2GTKI. In this study, patients in CP- CML treated with ponatinib had a better PFS and OS. This information should be considered with caution since data were pooled from the ponatinib PACE study and the EBMT registry to conduct an indirect comparison (59). Although this study included only patients harboring the T315 mutations, similar results (based on results from the PACE study) would be expected for patients without such mutations. Finally, in fit patients that experience disease progression to advanced phases and have an adequate donor, allogeneic stem cell transplantation remains the treatment of choice.

## Safety Considerations

All TKIs approved for the management of CML inhibit a range of kinases other than ABL, with such inhibition being associated with the development of side effects. In general, the most common side effects of TKI treatment in CML patients are cytopenias, nausea, diarrhea, fatigue, rash, and liver damage (Table 3) (13, 24, 60, 61). These side effects are frequent, but tend to be easily managed with dose reductions or transient drug interruption. However, low-grade toxicity (grade 1–2) can persist on time diminishing the QoL of patients. Other side-effects are unique or more specific to some TKIs, with the pathogenic mechanisms underlying such toxicity profile being incompletely understood (62). The main side-effects of each approved TKI are summarized below.

**Table 3:** Most frequent side effects related to treatment with tyrosine kinase inhibitors in CML patients^*^.

	**Imatinib**		**Dasatinib**		**Nilotinib**		**Bosutinib**		**Ponatinib**	
	**All grades**	**Grade 3/4**	**All grades**	**Grade 3/4**	**All grades**	**Grade 3/4**	**All grades**	**Grade 3/4**	**All grades**	**Grade 3/4**
Fatigue	++++	+	+++	+	++++	–	NR	NR	++++	++
Rash	++++	++	+++	+	++++	–	++++	++	++++	++
Headache	+++	–	++++	–	++++	–	++++	++	++++	++
Myalgia	+++++	–	++++	–	NR	NR	++	–	++++	++
Bone pain	+++	++	NR	NR	NR	NR	++	–	NR	NR
Diarrhea	++++	++	++++	+	+++	+	+++++	++++	NR	NR
Nausea	++++	–	++++	–	+++	+	++++	++	++++	+
Vomiting	+++	–	+++	–	++	–	++++	++	NR	NR
Abdominal pain	++	–	NR	NR	NR	NR	++++	++	++++	+++
Pancreatitis	+	+	NR	NR	++	++	NR	NR	+++	+++
Peripheral edema	++++	++	++++	++	+++	+	+++	++	NR	NR
Pleural effusion	++	+	++++	++	++	+	NR	NR	NR	NR
Elevated lipase	++++	+++	NG	–	++++	+++	++++	+++	++++	++++
Hepatotoxicity	++++	++	NG	+	+++++	+++	+++++	++++	+++	++
Anemia	+++++	+++	+++++	++++	++++	++	+++++	+++	++++	++++
Thrombocytopenia	+++++	++++	+++++	++++	++++	+++	+++++	++++	++++	++++
Neutropenia	+++++	++++	+++++	++++	++++	+++	++++	++++	++++	++++

* *This table has been adapted from J Apperley (60)*.

*Data derived from studies of first line use with the exception of ponatinib. + ≤ 1% of patients. ++ = 1–5%. +++ = 5–10%. ++++ = 10–50%. +++++ = 50–100%. NR, not reported; NG, data not given*.

### Imatinib

Imatinib is generally well-tolerated. Most frequent non-hematology side effects are gastrointestinal disturbances, edema, rash, and musculoeskeletal complaints. Imatinib therapy can rarely be associated with potentially irreversible acute renal injury (62). Imatinib long-term treatment may cause a clinically relevant decrease in the estimated glomerular filtration rate and hemoglobin levels that can improve after treatment discontinuation (63). No serious long-term exposure toxicity has been related to imatinib (1).

### Dasatinib

Around 30% of patients treated with dasatinib develop pleural effusion. It is important to know that this complication can occur anytime during treatment. It is usually well-managed with temporally interruptions, diuretics, and/or low dose steroids (64). However, many patients (~70%) experience a recurrence of the pleural effusion once dasatinib is restarted. Age and dasatinib dose are the main risk factors of pleural effusion (65). A recent study conducted by the MDACC group has shown a lower incidence of pleural effusion with adequate efficacy in patients treated with dasatinib 50 mg daily (15). The French group has also shown that dose adjustments based on dasatinib plasma levels can diminish pleural effusion rates while maintaining responses (66). For patients in risk of suffering pleural effusions, different strategies of dasatinib dose management can be proposed prior to the development of PE, such as daily dose reduction or, as an alternative option, an on/off treatment with a weekend drug holiday (67, 68). Pulmonary arterial hypertension (PAH) is an infrequent (occur in <1% of cases) but severe, and sometimes irreversible, complication of dasatinib treatment (69, 70).

### Nilotinib

Nilotinib treatment has been associated with an increased incidence of cardiovascular events. In the ENESTnd prospective randomized study, the incidence of cardiovascular events after 6 years was of 10% in patients treated with nilotinib 300 mg bid (5% ischemic heart disease, 1.4% ischemic cerebrovascular disease, and 4.3% peripheral arterial disease) as compared to 2.5% in imatinib treated patients (19). Risk factors for the occurrence of these complications are the nilotinib dose (higher with nilotinib 400 bid) and the presence of cardiovascular risks factors. Regarding this, it is important to be aware than an increasing plasma levels of glucose and cholesterol in patients treated with nilotinib have been described (62). However, nilotinib does not seem to induce diabetes mellitus, impared fasting glucose, or the metabolic syndrome when compare with imatinib or dasatinib (71).

In consequence, current experts' recommendations advocate against the use of nilotinib in patients with a high risk cardiovascular profile, whenever possible (62). Nilotinib is approved in second line treatment at a dose of 400 mg bid. Since such higher dose has been related to increased risk of cardiovascular events, the use of a lower dose (300 mg bid) in patients with intolerance to the first-line TKI seems reasonable.

### Bosutinib

Diarrhea is the most frequent side effect related to bosutinib in clinical trials (80% any grade, <10% of grade 3–4) This side-effect is usually well-managed with dose reductions/interruptions, since <1% of the patients treated with bosutinib in the BEFORE study discontinued treatment due to diarrhea (23, 24). In general, pleural effusion is a rare complication in bosutinib treated patients. However, patients who had pleural effusion while on dasatinib and later received bosutinib treatment had a high risk of recurrence of this complicacion (47). Cardiac and vascular toxicity have been evaluated in bosutinib treated patients enrolled in clinic al trials showing a good safety profile. Of interest, similar incidences were observed by bosutinib indication (first or later lines) or comparing to imatinib treated patients (72).

A decrease in the glomerular filtration rate has been observed with long-term exposure to bosutinib. Renal AEs were reported in 73/570 patients (13%) receiving second-line or later bosutinib, and in 22/248 (9%) receiving bosutinib first-line treatment (73).

### Ponatinib

The main concern with ponatinib is the increased incidence of cardiovascular complications that was observed in clinical trials using the approved dose of 45 mg/day. In the PACE trial, the cumulative incidence of arterial occlusive events in CP-CML patients was of 31% at 5 years (16% cardiovascular, 13% cerebrovascular, and 14% peripheral arterial vascular events) (52). Further data suggest that the vascular toxicity of ponatinib seems to be related with its dose and, therefore, dose reductions to 30 and 15 mg have been recommended as soon as patients achieve an optimal response (74). At this moment, there is a clinical trial evaluating the safety and efficacy of three ponatinib schemes starting doses of 45, 30, and 15 mg, with further doses modifications based on the degree of response. Hypertension was frequently observed in ponatinib treated patients (37% any grades, 14% of grade 3–4). Since hypertension was the most important risk factor for developing cardiovascular events, it should be aggressively treated in order to prevent such life-threatening complications (52). A recent study has shown that the Systematic Coronary Risk Evaluation could be a useful test to predict the risk of cardiovascular events in ponatinib treated patients. Finally, the beneficial effect of aspirin as primary thrombosis prophylaxis in this group of patients remains unsettled (75, 76).

## Emerging Treatment Options

### Radotinib

In a phase II trial, radotinib was effective and well-tolerated in patients with CP CML that did not respond to previous TKIs (95% of them had experienced imatinib failure). The rate of CCyR by 12 months was of 47%. OS and PFS rates at 12 months were of 96 and 86%, respectively. Radotinib was approved in Korea for the frontline treatment of CML based on the results of a phase III clinical trial that demonstrated its benefit in MMR rates as compared to imatinib (52 vs. 30% by 12 months). Of note, high rates of CCyR were found in both patient groups by 12 months: 91% with radotinib and 77% with imatinib (77).It is important to highlight that the trial was conducted in Asia, where higher response rates for TKIs therapy have previously been found (78, 79).

### Asciminib

Asciminib (ABL001) is a new BCR-ABL1 inhibitor that does not bind to the ATP-binding pocket where all the approved BCR-ABL1 TKIs do. In contrast, ABL001 binds to the myristoyl pocket of ABL1 and stabilizes the inactive conformation of the kinase. ABL001 was developed to test the hypothesis that dual inhibition of BCR–ABL1 using distinct targeting mechanisms might improve treatment outcomes by providing enhanced target coverage and preventing the emergence of resistance. In this line, crystallography studies showed that ABL001 and nilotinib can co-bind a single molecule of BCR–ABL1 and *in vitro* studies revealed additive effects of ABL001 in combination with imatinib, nilotinib, or dasatinib, with this preventing the emergence of resistance (80, 81). Preliminary results from an expanded phase I trial have demonstrated in a heavily pretreated CML population the significant efficacy of asciminib monotherapy with a good safety profile. Thus, 75% of patients achieved CCyR at 6 months of treatment, and some patients (4% of total) attained MMR at 12 months. Asciminib was well-tolerated, with the only grade 3–4 adverse events with probabilities >5% being lipase increase (10%) and thrombocytopenia (6%) (82). Recently, the results of ascimimib at a dose of 200 mg bid (higher that the 40 mg bid chosen for further development) in 32 patients harboring the T315I mutation have been presented. Remarkably, 80% of the patients achieved CCyR by 12 months of treatment. Results were even better in patients who had not previous exposure to ponatinib (83).

## Final Considerations

Currently, most patients diagnosed with CP CML will enjoy a survival expectancy of similar length than that of the general population. However, treatment success greatly depends on the judicious use of the available TKIs, since many patients will require sequential treatment due to inadequate response or side-effects. Thus, physicians attending CML patients should be familiar with the efficacy and toxicity profile of all five approved TKIs.

In the first line setting, the use of imatinib is an adequate treatment option for most patients considering its efficacy (low risk of progression and CML-related death), safety (good safety profile at the long-term), and price (relatively low with the generic formulation). However, in patients who may be willing to discontinue treatment (young patients or female candidates for pregnancy), the use of 2GTKIs upfront should be considered. In addition, patients with intermediate or high risk disease have a lower rate of transformation to advanced phases when treated with frontline nilotinib or dasatinib. Regardless of the first-line treatment, an early evaluation of the response with a swift treatment change in case of inadequate response is recommended.

For patients failing to imatinib, dasatinib, nilotinib, and bosutinib are appropriate treatment options. The selection of the second line treatment is based on the mutational BCR-ABL kinase domain status, the type of side effects related to imatinib (in order to avoid cross intolerance), and the patient's comorbidities (in order to avoid potential side effects). Nilotinib is not the best option for patients with uncontrolled cardiovascular risk factors. For such patients, dasatinib or bosutinib constitute a better choice. In patients with chronic obstructive pulmonary disease, cardiac insufficiency, or uncontrolled hypertension, TKIs other than dasatinib should be selected, whenever possible. Bosutinib is generally not the best treatment option in patients suffering gastrointestinal or liver disorders as well as in those with renal impairment.

A similar approach can be implemented in patients who develop intolerance to a 2GTKI. Treatment change in case of “warning response” according to the ELN may improve probabilities of obtaining optimal responses and DMR. However, treatment decision must take into consideration the potential side effects of the new TKI and the lack of evidence of an advantage in reducing disease progressions. Finally, in patients with resistance to 2GTKI the use of ponatinib is associated with the best response rates. To mitigate the risk of cardiovascular complications while on treatment, ponatinib dose should be reduced in responders and a proactive attitude toward controlling the vascular risk factors is highly recommended.

## Author Contributions

VG-G and JH-B wrote the manuscript.

### Conflict of Interest Statement

VG-G: Novartis: Speaker Honoraria, advisory committees; BMS: Speaker Honoraria, advisory committees; Pfizer: Speaker Honoraria, advisory committees. JH-B: Novartis: Speaker Honoraria, advisory committees; Incyte: Speaker Honoraria, advisory committees.
